# Mapping of major QTL and candidate gene analysis for hull colour in foxtail millet (*Setaria italica* (L.) P. Beauv.)

**DOI:** 10.1186/s12864-023-09517-9

**Published:** 2023-08-15

**Authors:** Shuqing Guo, Shaohua Chai, Yan Guo, Xing Shi, Fei Han, Ting Qu, Lu Xing, Qinghua Yang, Jinfeng Gao, Xiaoli Gao, Baili Feng, Hui Song, Pu Yang

**Affiliations:** 1 State Key Laboratory of Crop Stress Biology in Arid Areas, College of Agronomy, Northwest A & F University, Taicheng Road, Yangling, 712100 Shaanxi China; 2 Institute of Millet Crops, Anyang Academy of Agricultural Sciences, Anyang, 455000 Henan China

**Keywords:** Foxtail millet, Hull colour, Mixed inheritance analysis, QTL, Candidate genes

## Abstract

**Background:**

Hull colour is an important morphological marker for selection in seed production of foxtail millet. However, the molecular mechanisms underlying hull colour variation remain unknown.

**Results:**

An F_7_ recombinant inbred line (RIL) population containing 215 lines derived from Hongjiugu × Yugu18 was used to analyze inheritance and detect the quantitative trait loci (QTL) for four hull colour traits using major gene plus polygene mixed inheritance analysis and composite interval mapping (CIM) in four environments. Genetic analysis revealed that the hull colour L^*^ value (HCL^*^) was controlled by two major genes plus additive polygenes, the hull colour a^*^ value (HCa^*^) was controlled by three major genes, the hull colour b^*^ value (HCb*) was controlled by two major genes plus polygenes, and the hull colour C^*^ value (HCC*) was controlled by four major genes. A high-density genetic linkage map covering 1227.383 cM of the foxtail millet genome, with an average interval of 0.879 cM between adjacent bin markers, was constructed using 1420 bin markers. Based on the genetic linkage map and the phenotypic data, a total of 39 QTL were detected for these four hull colour traits across four environments, each explaining 1.50%–49.20% of the phenotypic variation. Of these, six environmentally stable major QTL were co-localized to regions on chromosomes 1 and 9, playing a major role in hull colour. There were 556 annotated genes within the two QTL regions. Based on the functions of homologous genes in *Arabidopsis* and the Kyoto Encyclopedia of Genes and Genomes (KEGG) and Gene Ontology (GO) gene annotations, five genes were predicted as candidate genes for further studies.

**Conclusions:**

This is the first study to use an inheritance model and QTL mapping to determine the genetic mechanisms of hull colour trait in foxtail millet. We identified six major environmentally stable QTL and predicted five potential candidate genes to be associated with hull colour. These results advance the current understanding of the genetic mechanisms underlying hull colour traits in foxtail millet and provide additional resources for application in genomics-assisted breeding and potential isolation and functional characterization of the candidate genes.

**Supplementary Information:**

The online version contains supplementary material available at 10.1186/s12864-023-09517-9.

## Introduction

Foxtail millet (*Setaria italica* (L.) P. Beauv.) is an important reserve cereal crop that is rich in proteins, vitamins, fatty acids, amino acids, and dietary fibers [1, 2]. Due to its unique nutritional and medicinal properties, the demand for foxtail millet has increased in recent years [3]. Hull colour is an important feature for evaluating the market value and appearance quality of foxtail millet [4–6]. The protein, lysine, fat, and selenium contents differ according to the hull colour of the foxtail millet [7].Cyan hull foxtail millet has high protein, fat, and lysine contents. Moreover, hull colour was related to bird feeding preferences during the mature phase of the foxtail millet crop. Hull colour results from the accumulation of plant pigments, including flavonoids, carotenoids, and betalains, which are plant-derived natural products known to have antioxidant activity and play an increasingly important role in food processing and healthcare [8–11]. Therefore, it is of great significance to explore the genetic factors responsible for hull colour variation in foxtail millet for studying the molecular mechanisms of regulating the synthesis and metabolism of flavonoids and lignin and further understanding its nutritional value.

Major gene plus polygene mixed inheritance analysis can be used to investigate the genetic characteristics of quantitative characters and to estimate the effect and variance in the major genes to preliminarily determine the genetic composition of breeding traits [12]. In recent years, major genes plus polygene genetic model has been widely used to analyze diverse agronomic traits in various plants, such as tomato internode length [13], related root traits in soybeans [14], and plant architecture traits in crape myrtles [15]. However, to the best of our knowledge, no previous studies have focused on the inheritance of hull colour using a major gene plus polygene genetic model in foxtail millet.

Quantitative trait loci (QTL) mapping has become increasingly crucial in molecular breeding that involves marker-assisted selection (MAS) and gene discovery [16]. In recent years, many researchers have used germplasm materials with various genetic backgrounds to construct segregated populations and applied them to the QTL mapping of important agronomic traits in foxtail millet. Regarding hull colour, several loci have been mapped to chromosomes 1, 2, 3, 6, 7, and 9 in foxtail millet [7, 17]. Xie et al. [2] showed that three QTL controlling hull colour are located on chromosome 1 of foxtail millet and identified a *cinnamyl alcohol dehydrogenase* (*CAD*) gene (*Seita.1G057300.v2.2*) as a candidate gene for the hull colour. Jia et al. [16] identified two QTL for hull colour on chromosomes 1 and 9 using genome-wide association studies in five environments. However, owing to its complex genetic basis, the molecular and genetic mechanisms underlying foxtail millet hull colour remain unclear. Futher studies are vital to improve the accuracy of QTL mapping and explore the genetic basis of hull colour traits in various environments.

In this study, a recombinant inbred line (RILs) population, YRRIL, derived from a cross between Hongjiugu and Yugu18, was developed and used to analyze the genetic laws according to mixed major gene plus polygene inheritance models. Additionally, We constructed a high-density linkage map. Based on the genetic linkage map and phenotypic data from the four environments, we sought to identify significant QTL associated with hull colour traits and predict candidate genes that are involved in hull colour change in foxtail millet. Related research could lay the foundation for further studies on the genetic basis of hull colour in foxtail millet.

## Results

### Phenotypic variation analysis for foxtail millet hull colour

The phenotypic characteristics of the parental lines and the YRRIL population were investigated in the four environments. The trait data indicated that HCL^*^ (hull colour L^*^), HCa^*^ (hull colour a^*^), HCb^*^ (hull colour b^*^), and HCC^*^ (hull colour C^*^) values were significantly different between the two parental lines (Table 1). The HCL^*^, HCb^*^, and HCC^*^ values of Yugu18 were significantly (*P* < 0.01) greater than those of Hongjiugu in all four environments. The HCa^*^ value of Yugu18 was lower than that of Hongjiugu (Table 1). The results indicated that the hull surface of Yugu18 was brighter than that of Hongjiugu and that Hongjiugu was redder than Yugu18. The HCL^*^, HCa^*^, HCb^*^, and HCC^*^ values of the YRRIL population displayed a wide range of variation, with coefficients of variation (CV) ranging from 6.70–41.41%. The HCL^*^ showed the smallest CV, while that of HCa^*^ was greater than 20% and showed relatively large variation ranges. This indicates that these traits have great potential for genetic improvement.

**Table 1:** Descriptive statistical results for hull colour of the parents and RIL population in four environments

Traits	Environments	Parents		YRRIL population					
		**Hongjiugu**	**Yugu18**	**Range**	**Mean ± SD**	**Kurtosis**	**Skewness**	**CV%**	***F***
HCL*	E1	47.50 ± 1.61b	61.27 ± 1.26a	40.00–64.91	51.87 ± 6.05	-1.01	-0.18	11.66	32.55^**^
	E2	51.66 ± 1.18b	63.86 ± 2.05a	48.92–70.10	56.63 ± 4.01	0.22	0.66	7.09	19.15^**^
	E3	48.58 ± 1.04b	60.98 ± 0.67a	46.83–63.10	53.95 ± 3.79	-0.86	0.21	7.02	22.68^**^
	E4	51.72 ± 0.82b	61.30 ± 2.03a	48.10–67.59	56.79 ± 3.81	-0.22	0.27	6.70	15.56^**^
HCa*	E1	15.31 ± 0.30a	6.08 ± 0.20b	5.25–22.86	11.62 ± 4.81	-0.67	0.74	41.41	71.43^**^
	E2	9.52 ± 0.58a	5.66 ± 0.68b	3.02–18.17	8.27 ± 2.31	0.91	0.50	27.97	13.45^**^
	E3	15.21 ± 0.21a	6.05 ± 0.11b	3.51–15.53	8.40 ± 2.85	-0.44	0.66	33.94	20.16^**^
	E4	9.77 ± 1.18a	6.17 ± 0.60b	3.23–16.50	8.35 ± 2.72	-0.31	0.47	32.58	18.61^**^
HCb*	E1	13.60 ± 1.98b	25.11 ± 1.34a	17.75–32.41	25.17 ± 3.11	-0.60	-0.24	12.37	10.82^**^
	E2	14.16 ± 2.01b	23.72 ± 1.31a	13.68–26.42	20.21 ± 2.78	-0.68	-0.02	13.77	8.72^**^
	E3	13.61 ± 1.96b	25.27 ± 1.12a	11.64–24.97	17.90 ± 2.71	-0.40	0.08	15.12	10.04^**^
	E4	15.39 ± 1.13b	22.94 ± 1.61a	13.92–25.54	19.81 ± 2.41	-0.55	-0.06	12.19	6.66^**^
HCC*	E1	14.60 ± 1.56b	26.11 ± 1.35a	22.10–34.98	28.18 ± 2.67	-0.36	0.20	9.48	7.24^**^
	E2	15.16 ± 3.01b	24.72 ± 2.31a	14.44–28.45	21.97 ± 2.72	-0.47	-0.10	12.37	7.30^**^
	E3	20.44 ± 1.48b	25.98 ± 1.11a	14.15–25.64	20.04 ± 2.15	0.07	0.00	10.73	26.20^**^
	E4	18.24 ± 1.58b	23.76 ± 1.63a	14.92–27.54	21.68 ± 2.33	-0.29	-0.20	10.76	24.55^**^

HCL*: hull colour L* value; HCa*: hull colour a* value; HCb*: hull colour b* value; HCC*: hull colour C* value; E1: 2020 in Yulin; E2: 2020 in Baoji; E3: 2021 in Yulin; E4: 2021 in Baoji; **: *p* ≤ 0.01

**Fig. 1:**
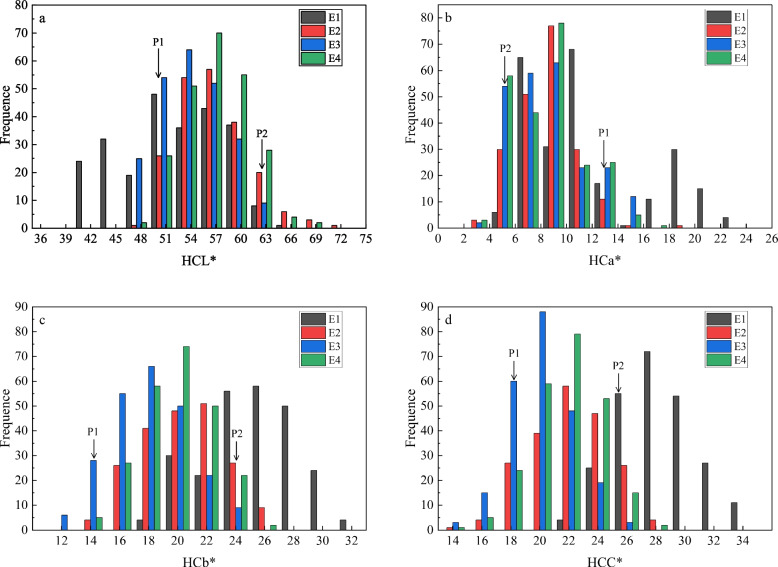
Frequency distribution of hull colour in YRRIL population. The P1 represents the Hongjiugu. The P2 represents the Yugu18

The values of HCL^*^, HCa^*^, HCb^*^, and HCC^*^ in the YRRIL population exhibited continuous variation and significant transgressive segregation, with values either larger or smaller than those of the parents in the four environments. The absolute values of skewness and kurtosis were < 1.0, suggesting that the traits were approximately normally distributed, and further hybrid genetic analyses could be performed. The frequency distribution showed that HCL^*^, HCa^*^ HCb^*^, and HCC^*^ showed some major peaks (Fig. 1), which indicated that hull colour might be controlled by the major genes and also influenced by minor genes.

### The major gene plus polygene mixed inheritance analysis for foxtail millet hull colour

Using a major gene plus polygene mixed inheritance analysis model, we investigated the inheritance patterns of the four hull colour traits in foxtail millet (Table 2).

**Table 2:** Analysis of the best models and genetic parameters for hull colour traits

Traits	Model	First-order parameter					Second-order parameter			
		**m**	**d**_**a**_	**d**_**b**_	**i**	**[d]**	***σ***_**mg**_^**2**^	***σ***_**pg**_^**2**^	***h***^**2**^_**mg**_** (%)**	***h***^**2**^_**pg**_** (%)**
HCL^*^	MX2-DE-A	59.05			-2.08	-6.05	7.74	4.51	50.31	29.31
HCa^*^	3MG-PEA	8.17	1.16	0.55			1.74		35.30	
HCb^*^	MX2-ED-A	17.92	-0.08	-3.01		-4.24	4.52	1.14	61.62	15.62
HCC^*^	4MG-EEEA	20.22	0.90	1.07			4.23		91.46	

HCL a*: hull colour L a* value; HCa a*: hull colour a a* value; HCb a*: hull colour b a* value; HCC a*: hull colour C a* value; *m* the population mean, *d*_*a*_ the additive effect of the first major gene, *d*_*b*_ the additive effect of the second major gene, *i* the epistatic effect of additive × additive between two major genes, *[d]* the additive effect of polygene, *σ*_*mg*_^*2*^ the genetic variance of the major gene, *σ*_*pg*_^*2*^ the genetic variance of multiple genes, *h*^*2*^_*mg*_ the heritability of the major gene, *h*^*2*^_*pg*_ the heritability of the multiple genes, *MX* mixed major gene plus polygene, *DE* duplicate effect, *A* Additive, *MG* major gene, *PEA* partially equally additive, *ED* epistatic dominance between two pairs of major genes, *EEEA* equally additive

The results indicated that the MX2-DE-A model (two pairs of duplicate effect major genes plus the additive polygenes model) was the most optimal genetic model for HCL^*^_._ These results suggested that HCL^*^ was controlled by two pairs of duplicate effect major genes plus additive polygenes. The epistatic effect from the interaction between two major genes was -2.08, and the additive effect among polygenes was -6.05. The heritability of the major genes and polygenes was 50.31% and 29.31%, respectively. The 3MG-PEA model (three pairs of partially equally additive major genes model) was deemed the most optimal genetic model for HCa^*^, suggesting that the trait was controlled by three pairs of partially equally additive major genes. The additive effects of the two major genes were 1.16 and 0.55, and the heritability of the major genes was 35.30%. The MX2-ED-A model was used to analyse the inheritance of HCb^*^. The results showed that HCb^*^ was controlled by two epistatic dominance major genes plus additive polygenes. The additive effects of the two major genes were -0.08 and -3.01, the additive effect among polygenes was -4.24, and the heritabilities of the major genes and polygenes were 61.62% and 15.62%, respectively. The 4MG-EEEA model (four pairs of partially equally additive major genes models) was deemed the most optimal genetic model for HCC^*^, suggesting that the trait was controlled by four pairs of major genes with three equally additive. The additive effects of the two major genes were 0.90 and 1.07, and the heritability of the major genes was 91.46%.

### Sequencing and SNP identification

**Fig. 2:**
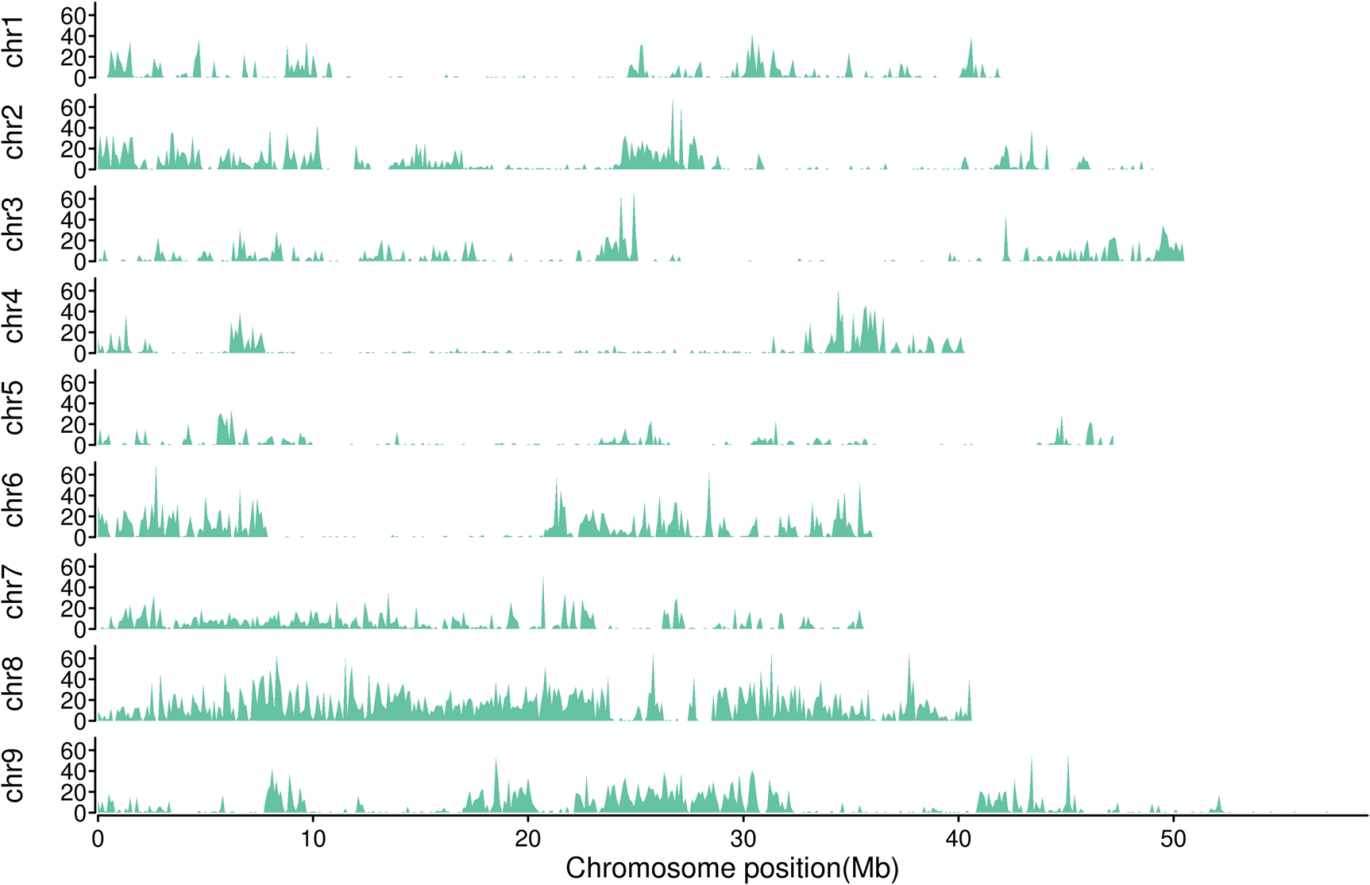
Distribution of the genetic variations on nine chromosomes of foxtail millet

Restriction-site associated DNA (RAD) sequencing of the parents generated two paired-end (PE) libraries with 150 bp reads on an Illumina HiSeq platform, including clean reads of approximately 617,473,058 bp in Hongjiugu and 659,941,345 bp in Yugu18. The clean base for the YRRIL population ranged from 33,493,575 to 3,399,626,258 bp with an average of 545,010,019 bp (Supplementary Table 1). The Q30 ratios (based on a quality score of 30, indicating a 1% chance of an error and thus 99% confidence) for the parents was 94.46% (Hongjiugu) and 94.58% (Yugu18), whereas, for the YRRIL population, they ranged from 93.98% to 97.21%, with an average of 94.82%. The guanine-cytosine (GC) contents was 48.72% and 48.05% in Hongjiugu and Yugu18, respectively. Similarly, the GC content of the YRRIL population ranged from 46.74% to 50.54%, with an average of 48.76% (Supplementary Table 1). The sequencing depths of the parental lines were approximately 17.63 × in Hongjiugu and 19.09 × in Yugu18 covering 6.85% and 6.99% of the whole genome, respectively. The sequencing depth for the YRRIL individuals ranged from 2.90 × to 53.66 × , with coverage between 2.51% and 14.07% (average of 6.48%) (Supplementary Table 2). By aligning the clean reads of the parental lines with the reference genome sequence of *Setaria italic* (JGI_v2.2), we obtained 20,748 SNP and 1,759 indels between the parents and the RIL. The number of SNP on each chromosome ranged from 843 on chromosome 5 to 5831 on chromosome 8 (Table 3 and Fig. 2).

**Table 3:** Summary of the bins and genetic variations distribution along nine chromosomes of foxtail millet

Linkage Group	Length (bp)	Number of SNPs	Number of InDels	Number of bins	Bin Interval (cM)	Genetic distance (cM)
Chr.1	42,145,699	1,314	139	154	0.973	148.894
Chr.2	49,200,776	2,446	218	164	0.950	154.928
Chr.3	50,652,576	1,608	168	151	0.901	135.220
Chr.4	40,408,058	1,274	135	161	0.804	128.615
Chr.5	47,253,416	843	78	130	1.150	148.308
Chr.6	36,015,257	2,453	225	150	0.734	109.401
Chr.7	35,964,515	1,846	150	147	0.861	125.677
Chr.8	40,690,061	5,831	440	183	0.531	96.704
Chr.9	58,970,518	3,133	206	180	1.004	179.635
Whole	401,300,876	20,748	1759	1420	7.908	1227.382

### A high-density genetic linkage map construction with bin marks

**Fig. 3:**
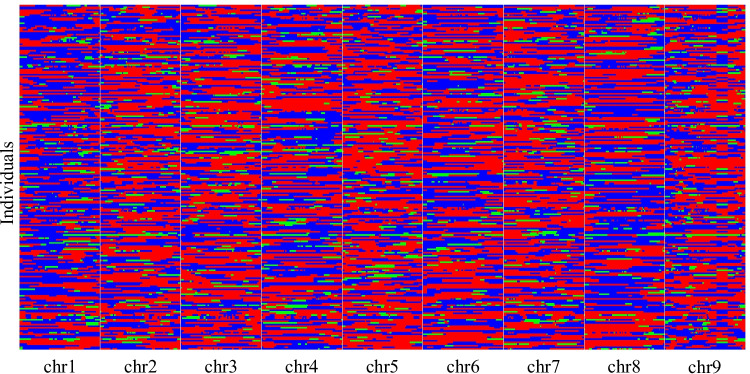
Recombination bin map of 215 foxtail millet RILs. The whole map contains 1420 bin markers. Red: genotype of Yugu18; blue: genotype of Hongjiugu; Green: heterozygous genotypes. Each row represents the genotype of an individual RIL chromosomes are separated by vertical white lines

**Fig. 4:**
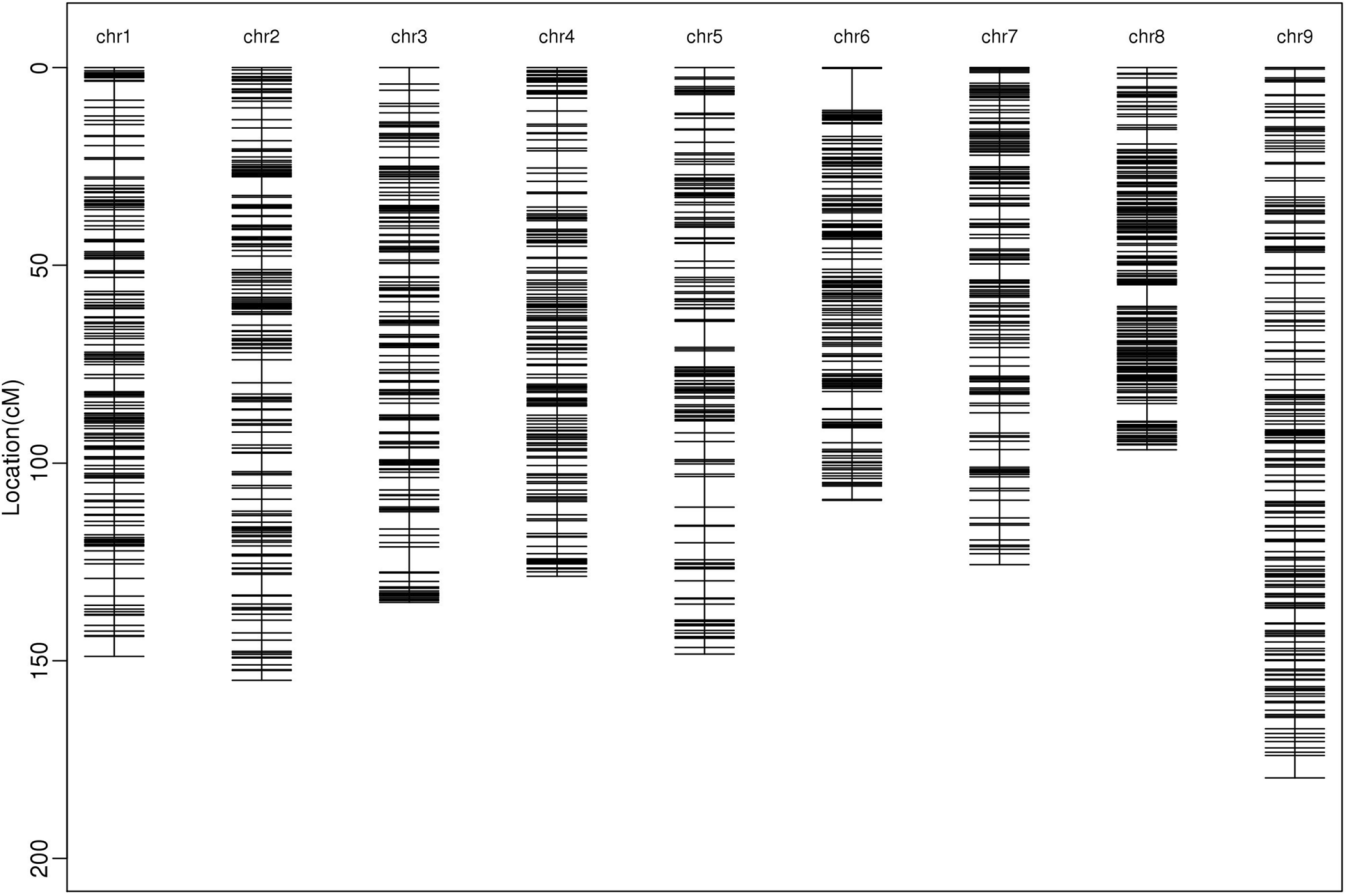
High-density linkage map of the foxtail millet YRRIL population based on the cross (Hongjiugu × Yugu18). The markers are indicated by black bars. The x-axis represents 9 linkage groups, and the y-axis represents the genetic distance

**Fig. 5:**
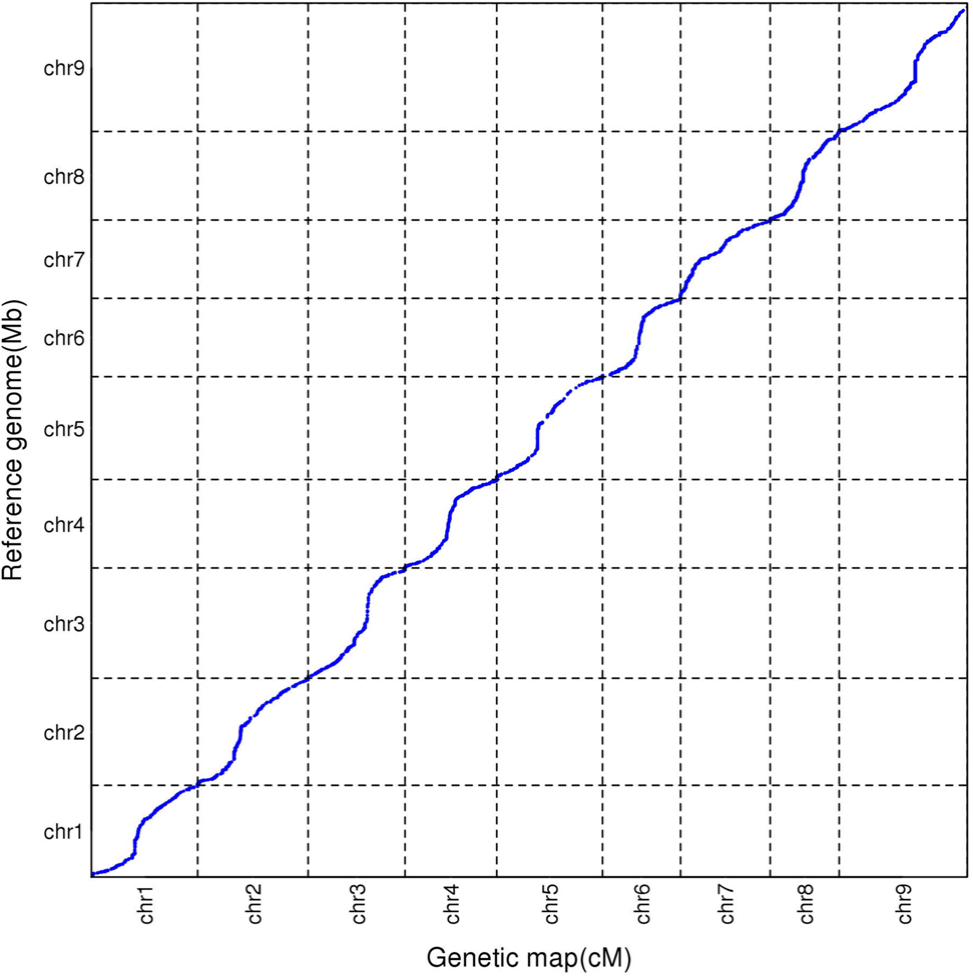
Collinearity map between genetic linkage map and reference genome. The x-axis represents the physical distance of each chromosome and y-axis represent the genetic length of each linkage group

In total 1,420 bin markers were detected using 20,748 SNPs in the YRRIL population (Table 3 and Fig. 3). These bin markers were used to construct a linkage map that spanned 1227.382 cM of the foxtail millet genome, with an average bin interval of 0.879 cM. The length of each linkage group ranged from 96.704 cM on chromosome 8 to 179.635 cM on chromosome 9. Genetic distances between the adjacent bin markers ranged from 0.531 cM–1.150 cM (Fig. 4 and Table 3). To evaluate the quality of the bin map built in the present study, a collinear graph of 1,402 bin markers between the genetic positions and their physical locations in the reference genome was plotted (Fig. 5). Our data showed that the genetic and physical positions of the bin markers corresponded approximately.

### QTL mapping analysis for foxtail millet hull colour

According to the high-density linkage map and the phenotypic data of the 215 individuals. A total of 39 QTL were detected for HCL^*^, HCa^*^, HCb^*^ and HCC^*^ on chromosomes 1, 2, 3, 4, 5, 6, 8, and 9 under four environments in the study (Table 4) and explained 1.50%–49.20% of the phenotypic variation, with LOD scores ranging from 3.38–44.61. Of 39 QTL, 13 were major QTL with the value of phenotypic variation greater than 10%. In addition, we also detected 26 QTL with medium or minor effects. These results indicate that this map contenting the QTL mapping requirements has wide applicability owing to its QTL detection efficiency.

**Table 4:** Summary of QTL for hull colour traits detected in the YRRIL population

Traits	Environments	QTL	Chromosome	Marker interval	Physical interval (bp)	LOD	Additive effect (%)	*R*^2^ (%)
HCL^*^	E1	*qHCL-1–1*	Chr.1	c01b039–c01b039	5470545–5553418	44.61	4.35	44.50
		*qHCL-1–2*	Chr.1	c01b051–c01b051	7988375–7988375	16.49	1.73	11.20
		*qHCL-2–1*	Chr.2	c02b124–c02b127	40839953–41868044	3.60	-0.76	1.50
		*qHCL-4–1*	Chr.4	c04b015–c04b020	1341692–1850065	3.55	-0.77	1.60
		*qHCL-6–1*	Chr.6	c06b068–c06b074	20111615–23692765	3.38	-0.75	1.50
		*qHCL-9–1*	Chr.9	c09b166–c09b167	49092452–49697847	11.59	1.85	8.80
		*qHCL-9–2*	Chr.9	c09b175–c09b175	54557509–54557509	29.86	2.71	19.00
	E2	*qHCL-1–1*	Chr.1	c01b038–c01b039	5392854–5553418	27.59	2.96	41.70
		*qHCL-5–1*	Chr.5	c05b116–c05b124	45252899–46230454	5.11	0.90	4.90
		*qHCL-9–3*	Chr.9	c09b008–c09b013	988753–1583761	8.65	-1.22	8.70
		*qHCL-9–2*	Chr.9	c09b175–c09b178	54557509–55252443	4.94	0.91	4.70
		*qHCL-9–1*	Chr.9	c09b167–c09b167	49697847–49697847	3.77	0.79	3.70
	E3	*qHCL-1–1*	Chr.1	c01b039–c01b039	5470545–5553418	36.65	2.69	45.70
		*qHCL-1–2*	Chr.1	c01b051–c01b051	7988375–7988375	10.42	1.71	17.20
		*qHCL-4–2*	Chr.4	c04b002–c04b012	540975–1101542	4.60	-0.69	3.30
		*qHCL-9–4*	Chr.9	c09b172–c09b173	52622280–53077613	8.25	1.01	7.00
	E4	*qHCL-1–1*	Chr.1	c01b039–c01b039	5470545–5553418	33.26	2.59	40.20
		*qHCL-4–3*	Chr.4	c04b160–c04b161	39756680–40248816	3.69	0.65	2.90
		*qHCL-8–1*	Chr.8	c08b092–c08b093	25649144–26168380	9.40	-1.09	7.80
		*qHCL-9–2*	Chr.9	c09b175–c09b176	54557509–54694792	8.35	1.05	7.20
HCa^*^	E1	*qHCa-1–1*	Chr.1	c01b039–c01b039	5470545–5553418	43.63	-3.20	37.60
		*qHCa-1–2*	Chr.1	c01b051–c01b051	7988375–7988375	11.93	-0.99	7.20
		*qHCa-9–1*	Chr.9	c09b175–c09b175	54557509–54557509	37.99	-2.49	25.20
		*qHCa-9–2*	Chr.9	c09b166–c09b167	49092452–49697847	13.04	-1.66	11.00
	E2	*qHCa-1–1*	Chr.1	c01b039–c01b039	5470545–5553418	33.13	-1.84	49.20
		*qHCa-1–2*	Chr.1	c01b051–c01b051	7988375–7988375	9.30	-0.49	9.30
		*qHCa-9–3*	Chr.9	c09b123–c09b127	41657680–42441674	3.61	0.49	3.10
		*qHCa-9–4*	Chr.9	c09b172–c09b172	52622280–52624644	7.31	-0.64	6.80
	E3	*qHCa-1–1*	Chr.1	c01b039–c01b039	5470545–5553418	31.81	-1.78	34.80
		*qHCa-1–2*	Chr.1	c01b051–c01b051	7988375–7988375	8.70	-0.58	6.20
		*qHCa-9–4*	Chr.9	c09b169–c09b172	50984590–52624644	11.70	-1.00	12.00
	E4	*qHCa-1–1*	Chr.1	c01b039–c01b039	5470545–5553418	37.41	-1.96	42.20
		*qHCa-9–1*	Chr.9	c09b176-c09b176	54694792–54694792	15.48	-1.03	13.10
		*qHCa-9–2*	Chr.9	c09b167–c09b167	49697847–49697847	5.63	-0.65	5.30
HCb^*^	E1	*qHCb-9–1*	Chr.9	c09b174–c09b175	53717341–54557509	15.82	1.66	25.10
		*qHCb-9–2*	Chr.9	c09b157–c09b159	47267844–47444954	5.58	1.05	9.90
	E2	*qHCb-3–1*	Chr.3	c03b035–c03b042	6358380–6883665	4.24	0.73	6.20
		*qHCb-6–1*	Chr.6	c06b022–c06b028	2469780–3417632	5.04	0.77	7.40
		*qHCb-9–3*	Chr.9	c09b011–c09b012	1334141–1450081	13.17	-1.33	21.60
		*qHCb-9–1*	Chr.9	c09b172–c09b177	52622280–54969651	4.55	0.74	6.90
	E3	*qHCb-1–1*	Chr.1	c01b027–c01b031	4079842–4574787	11.61	1.12	15.60
		*qHCb-1–2*	Chr.1	c01b154–c01b154	41595640–42080369	4.12	-0.01	4.90
		*qHCb-2–1*	Chr.2	c02b001–c02b015	14782–2221050	4.93	0.69	5.90
		*qHCb-4–1*	Chr.4	c04b008–c04b019	894459–1527105	4.04	-0.61	4.70
		*qHCb-9–1*	Chr.9	c09b173–c09b175	53010825–54557509	15.64	1.33	21.90
	E4	*qHCb-3–2*	Chr.3	c03b120–c03b121	45387254–45939137	6.52	0.75	9.40
		*qHCb-3–1*	Chr.3	c03b034–c03b044	6303987–7238657	4.36	0.62	6.50
		*qHCb-3–3*	Chr.3	c03b132–c03b134	47561089–48177283	4.05	0.61	6.00
		*qHCb-3–4*	Chr.3	c03b101–c03b105	38390280–40978876	3.78	0.57	5.60
		*qHCb-4–2*	Chr.4	c04b158–c04b160	39729882–39802274	3.83	0.60	6.20
		*qHCb-9–1*	Chr.9	c09b173–c09b176	53010825–54694792	8.68	0.95	15.10
		*qHCb-9–4*	Chr.9	c09b166–c09b167	49092452–49697847	3.60	0.62	6.60
HCC*	E1	*qHCC-1–1*	Chr.1	c01b039–c01b040	5470545–5690175	12.16	-1.27	19.60
		*qHCC-9–1*	Chr.9	c09b002–c09b016	526353–1983343	4.27	-0.69	6.10
	E2	*qHCC-3–1*	Chr.3	c03b108–c03b112	42510841–44275287	6.06	0.87	9.80
		*qHCC-3–2*	Chr.3	c03b035–c03b043	6358380–7210681	3.90	0.69	5.80
		*qHCC-6–1*	Chr.6	c06b032–c06b037	3556319–4075664	4.11	0.72	6.80
		*qHCC-9–1*	Chr.9	c09b011–c09b012	1334141–1450081	11.78	-1.29	21.50
	E3	*qHCC-9–1*	Chr.9	c09b007–c09b017	983209–2049720	5.40	-0.70	9.60
	E4	*qHCC-1–1*	Chr.1	c01b038–c01b039	5392854–5553418	6.30	-0.82	11.00
		*qHCC-3–3*	Chr.3	c03b116–c03b121	44780907–45939137	6.49	0.80	11.60

Thirteen QTL for HCL^*^ were detected on chromosomes 1, 2, 4, 5, 6, 8, and 9, and their phenotype variation ranged from 1.50%–45.70%. The* qHCL-1–1* was one of the 13 QTL in the physical interval of 5,392,854–5,553,418 bp on chromosome 1, which explained 40.20%–45.70% of the phenotypic variance across four environments and showed the greatest effects. The *qHCL-1–2* in the physical interval of 7,988,375–7,988,375 bp on chromosome 1 was detected in two test environments and explained 11.20%–17.20% of the phenotypic variation. The *qHCL-9–1* in the physical interval of 49,092,452–49,697,847 bp on chromosome 9, was detected in two test environments and explained 3.70%–8.80% of the phenotypic variation. The *qHCL-9–2* in the physical interval 54,557,509–55,252,443 bp on chromosome 9 were detected in three test environments and explained 4.70%–19.00% of the total phenotypic variations. The additive effects of the four QTL was derived from Hongjiugu.

Six QTL for HCa^*^ were detected on chromosomes 1 and 9, and the phenotype variation accounted by each of these seven QTL ranged from 3.10%–49.20%. Among these QTL, *qHCa-1–1* in the physical interval of 5,470,545–5,553,418 bp on chromosome 1 was mapped in four environments, which explained 34.80% to 49.20% of the phenotype variation, and showed the largest effects among the six QTL. The additive effect of *qHCa-1–1* was derived from Yugu18. *qHCa-1–2* in the physical interval of 7,988,375–7,988,375 bp on chromosome 1 and was mapped under three environments, explaining 6.20%–9.30% of the phenotype variation. The additive effect of *qHCa-1–2* came from Yugu18. *qHCa-9–1* in the physical interval of 54,557,509–54,694,792 bp on chromosome 9 and was mapped under two environments, explaining 13.10%–25.20% of the phenotype variation.*qHCa-9–2* in the physical interval of 49,092,454–49,697,847 bp on chromosome 9 was mapped under two environments, explaining 5.30%–11.00% of the phenotype variation. The additive effect of *qHCa-9–2* was derived from Yugu18. *qHCa-9–4* in the physical interval of 50,984,590–52,624,644 bp on chromosome 9 was mapped under two environments, explaining 6.80%–12.00% of the phenotype variation, and the additive effect of *qHCa-9–2* came from Yugu18.

Fourteen QTL for HCb^*^ were identified on chromosomes 1, 2, 3, 4, 6, and 9. The phenotype variation accounted for by each of these 14 QTL ranged from 4.70% to 25.10%. The *qHCb-9–1* in the physical interval of 52,622,280–54,969,651 bp on chromosome 9 was mapped under four environments, which explained 6.90%–25.10% of the phenotype variation and showed a relatively high effect. The additive effect of *qHCb-9–1* was derived from Hongjiugu. Major QTL *qHCb-9–3* in the physical interval of 1,334,141–1,450,081 bp on chromosome 9 explained 21.26% of the phenotype variation, and the additive effect of *qHCb-9–3* was derived from Yugu18. Major QTL *qHCb-1–1* in the physical interval of 4,079,842–4,574,787 bp on chromosome 9 explained 15.60% of the phenotype variation.

Six QTL for HCC^*^ were identified on chromosomes 1, 3, 6, and 9. The phenotype variation accounted for by each of these six QTL ranged from 5.80% to 21.50%. The *qHCC-1–1* in the physical interval of 5,392,854–5,690,175 bp on chromosome 1 was mapped under two environments, explaining 11.00%–19.60% of the phenotype variation. The additive effect of *qHCC-1–1* came from Yugu18. The *qHCC-9–1* in the physical interval of 5,263,53–2,049,720 bp on chromosome 9, was mapped under three environments, explaining 6.10%–21.50% of the phenotype variation The additive effect of *qHCC-9–1* was derived from Yugu18. Major QTL *qHCC-3–3* in the physical interval of 44,780,907–45,939,137 bp on chromosome 3, explaining 11.60% of the phenotype variation, and the additive effect of *qHCC-3–3* was derived from Hongjiugu.

### QTL analysis with epistatic interaction effects for the foxtail millet hull colour

Epistatic interactions were evaluated using QTLNetwork (version 2.0). A total of six pairs of significant epistatic QTL were detected, explaining 0.01%–2.76% of the phenotypic variance for hull colour, and the epistatic effects of the six pairs of QTL ranged from -0.71–0.73 (Table 5). Of these, two pairs of major QTL showed significant epistatic effects. The genome of the environmentally stable and major QTL *qHCa-1–1* overlapped with the *qHCb-1–3*, and both significantly interacted with the major QTL *qHCa-9–1*, explaining 2.76% and 0.78% of the phenotypic variance in hull colour, respectively. Moreover, *qHCb-1–3* interacts with *qHCb-8–1* and *qHCb-8–2*. Other environmentally stable major QTL were not involved in epistatic interactions across the four environments, indicating that foxtail millet hull colour can be regulated by the additive effects of these major QTL. No significant epistatic × environment interaction effects were observed among the major QTL.

**Table 5:** Summary of the epistatic and epistatic × environment interaction effect of QTL associated with hull colour traits in the YRRIL population

Traits	QTL_i_	Marker interval	QTL_j_	Marker interval	Epistatic effect (AA)		Epistatic × environment interaction effect				
					**AA**	***h***^**2**^**/%**	**AA-E1**	**AA-E2**	**AA-E3**	**AA-E4**	***h***^**2**^**/%**
HCa ^a^	*qHCa-1–1*	c01b039–c01b040	*qHCa-9–1*	c09b174–c09b175	0.73 ^c^	2.76	0.64 ^c^	-0.28 ^a^	-0.12	-0.24	0.90
HCb ^a^	*qHCb-1–3*	c01b038–c01b039	*qHCb-8–1*	c08b067–c08b068	0.36 ^c^	1.09	0.00	0.00	-0.00	-0.00	0.10
	*qHCb-1–3*	c01b038–c01b039	*qHCb-8–2*	c08b091–c08b092	0.16 ^a^	0.01	0.00	0.00	-0.00	-0.00	0.02
	*qHCb-1–3*	c01b038–c01b039	*qHCb-9–1*	c09b175–c09b176	-0.71 ^c^	0.78	-0.64 ^c^	0.19	0.30 ^a^	0.15	0.47
HCC ^a^	*qHCC-4–1*	c04b158–c04b159	*qHCC-9–2*	c09b093–c09b094	0.22 ^b^	1.24	-0.00	0.00	-0.00	-0.00	0.16
	*qHCC-4–2*	c04b158–c04b159	*qHCC-9–3*	c09b102–c09b103	0.27 ^c^	0.01	-0.00	0.00	0.00	-0.00	0.08

*AA* the epistatic effect, *h*^*2*^ the heritability of the epistatic effect, AA-E1: AA-E1 indicates the epistatic × environment interactiont effec in the E1; AA-E2: the epistatic × environment interactiont effec in the E2; AA-E3: the epistatic × environment interactiont effec in the E3; AA-E4: the epistatic × environment interactiont effec in the E4; *h*^2^: the heritability of the epistatic × environment interaction effect

^a^ The significances at the probability level of 0.05

^b^ The significances at the probability level of 0.01

^c^ The significances at the probability level of 0.005

### Prediction of candidate genes in two major QTL intervals for foxtail millet hull colour

**Fig. 6:**
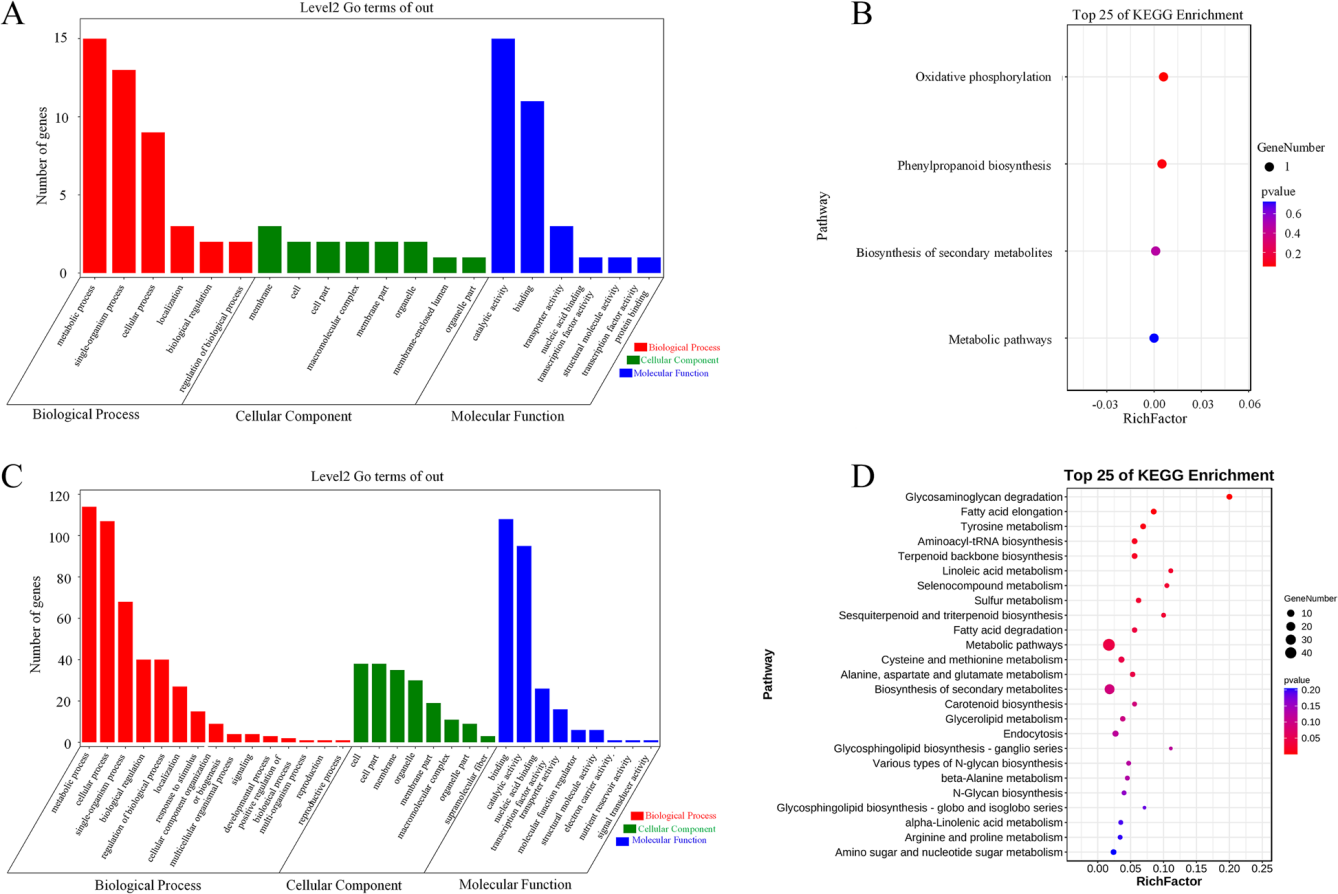
GO and KEGG enrichment of major QTL region genes. **A** GO enrichment in the 5,392,854–5,553,418 bp region of chromosome 1. **B** KEGG enrichment in the 5,392,854–5,553,418 bp region of chromosome 1 (The metabolism pathways were retrieved from the KEGG database: www.kegg.jp/kegg/kegg1.html). **C** GO enrichment in the 52,622,280–54,969,651 bp region of chromosome 9. **D** KEGG enrichment in the 52,622,280–54,969,651 bp region of chromosome 9 (The metabolism pathways were retrieved from the KEGG database: www.kegg.jp/kegg/kegg1.html)

Functional annotation of genes in major and stable QTL regions helps to reveal their biological functions. The stable major QTL *qHCL-1–1* overlapped with the major QTL *qHCa-1–1* and *qHCC-1–1* in an interval of 5,392,854–5,553,418 bp on chromosome 1. Based on the foxtail millet reference genome (JGI_v2.2), 47 genes were annotated in this genomic region (Supplementary Table 3). Significantly enriched biological processes were identified by GO enrichment analysis of these genes..The GO enrichment results included biological process (BP), cellular component (CC), and molecular function (MF), with the largest number of functional classifications was annotated into CC, accounting for eight categories. Most of the enriched genes participated in the membrane, cell, cell part, macromolecular complex, membrane part, and organelle. In the BP classification, the annotated genes were mostly enriched in metabolic, single-organism, and cellular processes. Most of the clustered genes in the MF classification were clustered into catalytic activity and binding (Fig. 6A). To further determine the metabolic pathways associated with hull colour, Kyoto Encyclopedia of Genes and Genomes (KEGG) enrichment analysis was performed (Fig. 6B). Oxidative phosphorylation, phenylpropanoid biosynthesis, biosynthesis of secondary metabolites, and metabolic pathways were the top 25 significantly enriched pathways. Based on the annotation information and functions of homologous genes in *Arabidopsis*, three potential candidate genes that may be directly or indirectly related to hull colour were predicted. *Seita.1G057300.1* occurred in the phenylpropanoid biosynthesis (pathway_idKO00940), biosynthesis of secondary metabolites (pathway_idKO01110), and metabolic pathways (pathway_idKO01100). The *Arabidopsis* homolog of *Seita.1G057300.1,* AT3G19450, encodes a catalytically active cinnamyl alcohol dehydrogenase that acts as the primary gene involved in lignin biosynthesis in the floral stem of *Arabidopsis thaliana* by supplying both coniferyl and sinapyl alcohols. *Seita.1G059100.1* and *Seita.1G059300.1* occurred in the oxidation–reduction process (GO:0055114, GO:0020037, GO:0016705, and GO:0005506). The *Arabidopsis* homolog of *Seita.1G059100.1* and *Seita.1G059300.1* is *AT5G07990*, which encodes the Cytochrome P450 superfamily protein required for flavonoid 3' hydroxylase activity (Table 6).

**Table 6:** Detailed information of five candidate genes related to hull colour traits

Traits	QTL	Chromosome	Gene ID	K0	GO	Homologous genes in *Arabidopsis*	Functional annotation
Hull colour	*qHCL-1–1* *qHCa-1–1* *qHCC-1–1*	Chr.1	*Seita.1G057300.1*	K00083	GO:0055114, GO:0016491, GO:0008270	*AT3G19450.1*	GroES-like zinc-binding alcohol dehydrogenase family protein
			*Seita.1G059100.1*		GO:0055114, GO:0020037, GO:0016705, GO:0005506	*AT5G07990.1*	Cytochrome P450 superfamily protein
			*Seita.1G059300.1*		GO:0055114, GO:0020037, GO:0016705, GO:0005506	*AT5G07990.1*	Cytochrome P450 superfamily protein
	*qHCb-9–1 qHCL-9–2* *qHCa-9–1*	Chr.9	*Seita.9G515900.1*	K02293		*AT4G14210.1*	phytoene desaturase 3
			*Seita.9G515900.2*	K02293		*AT4G14210.1*	phytoene desaturase 3

Another environmentally stable and major *qHCL-9–2* shared an overlapping QTL genetic region (52,622,280–54,969,651 bp) on chromosome 9 with the stable and major QTL, *qHCa-9–1* and *qHCb-9–1*, covering 509 annotated genes. GO enrichment showed that the largest number of functional classifications were annotated into BP, accounting for 15 categories, of which most of the enriched genes participated in metabolic, cellular, and single-organism processes. In the CC classification, the annotated genes were mostly enriched in the membrane, cell, cell parts, membrane, and membrane parts, indicating their localisation in the cell membrane. Most clustered genes in the MF classification were clustered into binding, catalytic, and nucleic acid binding transcription factor activities (Fig. 6C). Metabolic pathways, biosynthesis of secondary metabolites, and carotenoid biosynthesis were found in the top 25 significantly enriched pathways based on the KEGG enrichment results (Fig. 6D). Two homologous genes have been predicted in *Arabidopsis*. *Seita.9G515900.1* and *Seita.9G515900.2* were located in the KEGG pathway (pathway_idK02293) associated with carotenoid biosynthesis. The Arabidopsis homolog of *Seita.9G515900.1* and *Seita.9G515900.2* is *AT4G14210*, which encodes phytoene desaturase (phytoene dehydrogenase), which catalyses the desaturation of phytoene to zeta-carotene during carotenoid biosynthesis. These results indicated that the five genes responsible for the biosynthesis of secondary metabolites in these QTL regions were very likely to be causal genes controlling hull colour traits.

## Discussion

### Application of genetic model analysis in foxtail millet breeding

Hull colour is an important character closely related to seed quality [18, 19]. For foxtail millet, those with redder and more yellow hulls are of higher quality and have a promising applications [20]. Few reports on the genetic basis of hull colour of foxtail millet have severely restricted the promotion of high-quality foxtail millet. In this study, we obtained the F_7_ RIL population from two parents with contrasting hull colours and investigated the phenotypic variations and genetic architecture of hull colour in a population of foxtail millet grown in four environments. The results showed that the four hull colour traits were approximately normally distributed, corresponding to the genetic characteristics of a mixed major gene plus polygene inheritance model. Genetic analysis indicated that both HCL^*^ and HCb^*^ were controlled by two major genes plus additive polygenes. Unlike the other two characteristics, HCa^*^ was controlled by three major genes, and HCC^*^ was controlled by four major genes, respectively. The heritability of the major genes of these traits was greater than that of the polygene, indicating that hull colour was mainly controlled by the major gene and was less affected by the environment. Therefore, it is relatively easy to select hull colours in early generations of breeding programs [13]. It is necessary to identify QTL associated with hull colour through further experiments to help guide the molecular-assisted breeding of foxtail millet to improve breeding efficiency.

### High-density genetic map enables to narrow down QTL region and identify environmentally stable major QTL

A genetic map is fundamental for understanding the genetic basis of the foxtail millet genome and for identifying genes related to important agronomic traits. Insufficient molecular markers and the lack of a high-throughput genotyping platform limit map-based cloning of foxtail millet. In the current study, 1420 bin markers developed from RAD sequencing were used to construct a high-density genetic map covering 1227.382 cM, with an average interval of 0.879 cM between adjacent bin markers.

A total of 39 QTL for four hull colour-related traits were identified on chromosomes 1, 2, 3, 4, 5, 6, 8, and 9 by developing a high-density genetic map and phenotypic data measured in the RIL population. 13 were major QTL explaining more than 10% of the phenotypic variation. Three major QTL were associated with GCL^*^ (*qHCL-1–1*, *qHCL-1–2*, and *qHCL-9–2*), four major QTL were associated with GCa^*^ (*qHCa-1–1*, *qHCa-9–1, qHCa-9–2* and *qHCa-9–4*), three major QTL were associated with GCb^*^ (*qHCb-1–1*, *qHCb-9–1*, and *qHCb-9–3*), and three major QTL were associated with GCC^*^ (*qHCC-1–1*, *qHCC-9–1*, and *qHCC-3–3*). Nine of the 13 major QTL identified were detected in at least two environments and thus were considered as stable QTL in this study. The LOD and R^2^ values for the six QTL were particularly high, and these QTL were the most compelling for marker development.

Hull colour has been reported to be mainly controlled by a few major QTL on chromosomes 1, 2, 3, 6, 7, and 9 [2, 7, 13, 21]. We compared the genomic regions of the QTL identified in this study with those detected in previous studies. Xie et al. [2] detected and narrowed the QTL regions of hull colour into 70 kb (5.43–5.50 Mb) and 30 kb (5.69–5.72 Mb) intervals on chromosome 1. Jia et al. [13] identified the QTL for hull colour in the interval of 1–5,480,917 bp (1–5,467,847 bp and 1–5,476,983 bp) on chromosome 1 by genome-wide association studies from five locations. Tian et al. [7] identified the QTL for hull colour in the interval of 4,570,517–10,698,955 bp on chromosome 1 by the bulk segregant analysis-RNA sequencing (BSR-Seq) approach, which partially overlapped with *qHCL-1–1* (5,392,854–5,553,418 bp), *qHCa-1–1* (5,470,545–5,553,418 bp) and *qHCC-1–1* (5,392,854–5,690,175 bp) identified in our research. Compared to the genomic region previously identified, the genomic region for QTL was narrowed down to smaller intervals in the current study, which allowed us to further narrow down the selection of plausible causal genes [16]. Jia et al. [13] identified another QTL for hull colour in the interval of 1–54,490,294 bp (1–54,533,849 bp or 1–54,490,656 bp) on chromosome 9, which was adjacent to the locus *qHCL-9–2* (54,557,509–55,252,443 bp), *qHCa-9–1* (54,557,509–54,694,792 bp), and *qHCb-9–1* (54,622,280–54,969,651 bp) in our research. There was a small physical distance between them, which might be because they were likely to be at the same locus. In addition, the minor QTL *qHCL-2–1* (40,839,953–41,868,044) and *qHCb-3–3* (*47,561,089*–*48,177,283*) overlapped with those QTL that Tian et al. [7] identified.

In summary, the major QTL *qHCL-1–1, qHCa-1–1*, *qHCC-1–1*, *qHCL-9–2*, *qHCa-9–1*, and *qHCb-9–1* were more stable and had large effects. We hypothesized that the regions of these QTL contain key loci affecting hull colour in foxtail millet, which might provide a good foundation for further research on traits related to hull colour at the molecular level.

### QTL cluster and candidate genes

In the process of QTL mapping, one or more QTL in the same or similar positions may simultaneously affect multiple traits, showing the phenomenon of clustering distribution [22]. A total of two QTL clusters were identified in this study, which contained six stable QTL, namely *qHCL-1–1, qHCa-1–1*, *qHCC-1–1*, *qHCL-9–2*, *qHCa-9–1*, and *qHCb-9–1*. There were 556 genes annotated in the two significant intervals based on the foxtail millet reference genome, of which five were predicted as candidate genes combined with the function of homologous genes in *Arabidopsis* in previous studies. This could be a valuable resource for gene cloning and understanding the genetic basis of hull colour.

Recent studies on rice and maize have shown that many genes related to hull colour traits are involved in the metabolic pathways of flavonoids and lignin [23, 24]. In this study, we identified five candidate genes associated with lignin, flavonoid, and carotenoid biosynthesis in the genomic regions on chromosomes 1 and 9. *Seita.1G057300.1* was annotated as a GroES-like zinc-binding alcohol dehydrogenase family protein that encodes a catalytically active cinnamyl alcohol dehydrogenase and acts as the primary gene involved in lignin biosynthesis in *Arabidopsis thaliana* [25]. Interestingly, the gene *Seita.1G057300.1* was detected in previous studies [2], suggesting that our strategy was effective. Zhang et al. [26] found that *gh2* encodes cinnamyl alcohol dehydrogenase (CAD), and the *gh2* mutant is lignin-deficient mutant. Further analysis revealed that a single base mutation (G to A) at the 3563 bp site of *CAD* gene in the *gh881* mutant, which led to a G297D mutation (codon GGC to GAC). Mutations in the *CAD* genes also caused a reddish-brown colour in the leaf midribs and stemmed sclerenchyma in Zea. *mays*, brown vascular tissue, and altered lignin content in S. *bicolor* [27]. Hirano et al. [28] found that *OsCAD2* is the major *CAD* gene responsible for monolignol biosynthesis in rice culms. Xie et al. [2] found the CAD (*Seita.1G057300.1*) possessed allelic variations in the coding region and amino acid changes between two parental lines by mapping the whole-genome resequencing reads of the two parent lines to the reference genome and calling SNPs and indels. Therefore, the gene *Seita.1G057300.1* warrants further verification as a candidate gene regulating hull colour using genomic editing and transgenic approaches.

*Seita.1G059100.1* and *Seita.1G059300.1* were annotated as a cytochrome P450 superfamily protein, which is required for flavonoid 3' hydroxylase (F3'H) activity in *Arabidopsis thaliana* [29]. F3'H is one of the key enzymes in anthocyanin biosynthesis that has been identified in a variety of ornamental plants [30]. *Seita.9G515900.1* and *Seita.9G515900.2* encode phytoene desaturase 3 (PDS3), which participates in catalyzing the desaturation of phytoene to zeta-carotene during carotenoid biosynthesis as a key enzyme in *Arabidopsis thaliana*. Qin et al. [31] confirmed that both the albino and dwarf phenotypes of the pds3 mutant resulted from functional disruption of PDS3 and found that chloroplast development was arrested at the proplastid stage in the pds3 mutant. Zhao et al. [32] showed that the yellowish-white flower (ywf) mutant exhibited lower carotenoid content with a reduced and defective chromoplast ultrastructure in the petals of *Brassica napus*. Genetic analysis has revealed that YWF, which encodes phytoene desaturase 3 (PDS3), is involved in carotenoid biosynthesis.

In the present study, the key loci on chromosome 1 were also detected in previous studies [2]. In this region, a cinnamyl alcohol dehydrogenase (*CAD*) gene was identified as a candidate gene. New genes that are important for the metabolic pathways of flavonoids, carotenoids, and lignins in foxtail millet were predicted. 

## Conclusions

This study serves as a reference for hull colour inheritance and molecular investigation. A set of 215 lines derived from a cross between Hongjiug and Yugu18 were used to analyze the inheritance and detect QTL for hull colour traits in this study. Inheritance analysis shows that HCL^*^ was controlled by two major genes plus polygenes. HCa^*^ was controlled by three major genes, HCb^*^ was controlled by two major genes plus polygenes, and HCC^*^ was controlled by four major genes. We constructed a high-density genetic linkage map for a novel interspecific RIL population that enabled the genetic dissection of the hull colour trait in foxtail millet. 39 QTL for HCL^*^, HCa^*^, HCb^*^, and HCC^*^ were mapped to chromosomes 1, 2, 3, 4, 5, 6, 8, and 9. Six major QTL were repeatedly detected in at least two environments with particularly high LOD and R^2^ values. Five candidate genes were predicted in the overlapping areas of the six QTL. This work serves as a reference for foxtail millet hull colour inheritance and molecular investigation and provides a foundation for further research on hull colour genetic regulation and molecular breeding.

## Material and methods

### Plant materials

**Fig. 7:**
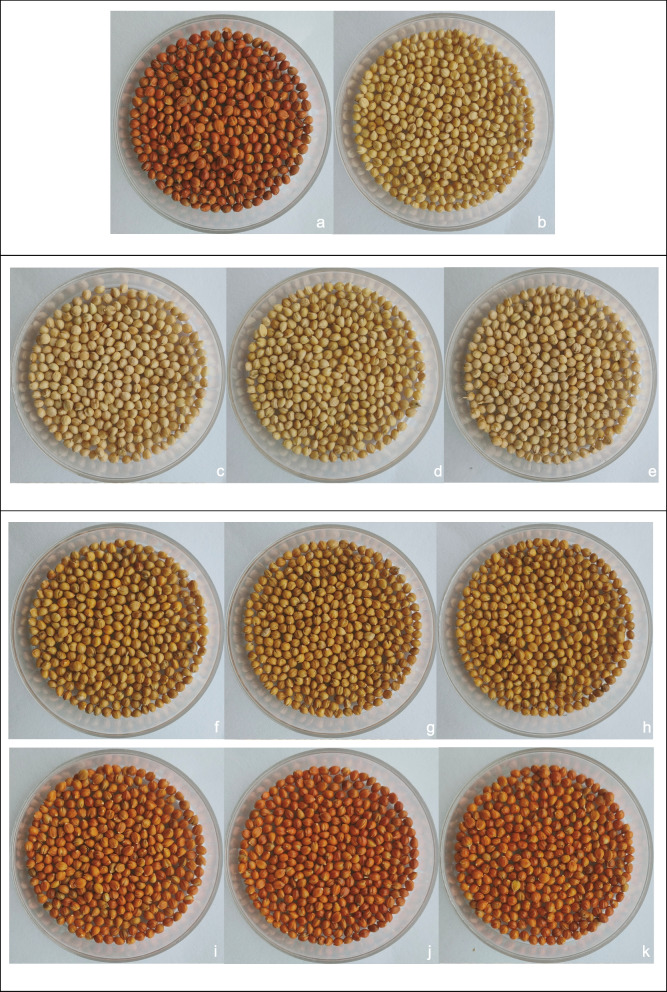
Images of Hongjiugu, Yugu18 and the different colour variants in the RIL population **a** Hongjiugu (female parent); **b** Yugu18 (male parent); **c** YRRIL-1; **d** YRRIL-63; **e** YRRIL-158; **f**  YRRIL-141; **g** YRRIL-142; **h** YRRIL-194; **i** YRRIL-8; **j** YRRIL-10; **k** YRRIL-125

Two foxtail millet varieties, Hongjiugu (female parent) and Yugu18 (male parent) (Fig. 7), and an F_7_ recombinant inbred lines (RILs) population, named YRRIL, comprising 215 lines derived from a cross between these two varieties, were used in this study. For the field experiment, two parents and the RILs population were planted in a completely randomized block with three replicates at two experimental sites in Yulin city (37°56^′^N, 109°21^′^E) and Baoji city (34°26^′^N, 107°37^′^E), Shaanxi province, China, in the growing season of the year 2020–2021. At the beginning of the field experiment, the entire field was ploughed to mix the soil thoroughly and harrowed to ensure uniform soil conditions in each experimental plot. The field was divided into three repeated blocks. A total of 215 lines were randomly planted in each block. In every plot, each line was planted in four rows with 2 m in length and with 0.30–0.40 m between rows. Plants were grown under standard agronomic practices.

### Phenotypic data collection

All seeds were harvested at physiological maturity. Three well-filled foxtail millet panicles per plot were collected from each plot. A colorimeter (Ci7600, USA) was used to quantify hull colour (illuminant D65, illumination area diameter 10 mm). The apparatus calculates and returns the colour parameters from the spectra. The colorimeter was calibrated relative to the white and black references before beginning the measurements and again every 15 min while the measurements were conducted. The visual colour of the samples was determined after placing mature seeds in their respective packs. Whole samples were placed on a white background and the colour values L^*^, a^*^, and b^*^ were measured at three random positions on each sample, with three biological replicates. The parameter L^*^ represents the measure of the degree of lightness, with values ranging from 0 to 100 (i.e., black = 0 and white = 100); parameter a^*^ represents red to green colouration, where a positive value represents the colour progression towards red and a negative value towards green; and parameter b^*^ represents blue to yellow colouration, where a positive value refers to more yellowish and negative value towards blue colouration. The parameter C^*^, which represents the colour saturation, is calculated as follows:

$${\mathrm{C}}^{{*}_{2}}={\mathrm{a}}^{{*}_{2}}+{\mathrm{b}}^{{*}_{2}}$$

### Major gene plus polygene mixed inheritance analysis

A mixed major gene plus polygene inheritance model was used to analyze a dataset of hull colour traits in foxtail millets from the YRRIL population and parental lines [33]. Component parameters were estimated using the maximum likelihood method based on the iterated expectation and conditional maximization (IECM) algorithm. According to Akaike’s Information Criterion (AIC), the models with smaller AIC values were considered the candidate genetic models. Fitness tests (*U*_*1*_^*2*^*, U*_*2*_^*2*^*, U*_*3*_^*2*^*, nW*^*2*^, and *Dn*) were performed on the candidate genetic models. The model with the minimum number of values below the statistical significance level was selected as the best optimal model. Finally, the genetic parameters of the optimal model were estimated using the least-squares method. The R software SEA [34, 35] was used in this study.

### Sequencing of parental lines and RIL population

Young leaf tissues from the two parental lines and YRRIL inbred lines were collected to extract total genomic DNA using the CTAB method [36]. DNA degradation and contamination in all lines were monitored using 1% agarose gel electrophoresis. A NanoDrop UV–Vis spectrophotometer and Qubit 3.0 were used to check and measure DNA purity and concentration, respectively. The DNA concentration and the total DNA amount per sample were greater than 40 ng/µL and 2 µg, respectively. Sequencing libraries were constructed using the NEBNext® Ultra™ II DNA Library Prep Kit for Illumina®. The constructed libraries were sequenced using the Illumina HiSeq platform, and 150 bp paired-end reads were generated. Low-quality reads with adaptor sequences and duplicated reads were filtered using Cutadapt (version 1.13) and Trimmomatic (version 0.36), and the remaining high-quality data were used for further bioinformatics analysis.

### Sequence alignment, genotyping, and recombination breakpoint determination

Reads from all samples were aligned to align with the reference genome sequence of *Setaria. italica* (JGI_v2.2) using the BWA software (version 0.7.15-r1140). The alignment files were converted into BAM files using samtools software (version 1.3.1) [37]. After alignment, the SNPs and indels of all samples were identified using the GenotypeGVCFs of the Genome Analysis Toolkit (GATK) Software (version 3.7) [38]. Only SNP markers that were homozygous in both parents and polymorphic between the parents were used for further analyses. The variations with a miss rate of more than 80% and heterozygosity of more than 15% were discarded before the linkage analysis. The SNP annotation of the reference genome was performed using the ANNOVAR package (version 2016Feb1).

### Construction of the genetic linkage map and QTL mapping

To ensure a high-quality genetic map of the RIL population, recombination frequencies between markers were calculated using MSTMap and converted into genetic distances using the Kosambi algorithm [39, 40]. The phenotype of each RIL and the genotype of each bin was collected for QTL analysis. QTL Cartographer (version 1.17j) software was used to detect QTL using the Composition Interval Mapping (CIM) mapping method. Each group of phenotypic data was iterated 1,000 times to calculate the *P*-value, and QTL were considered for LOD > 3.0. In addition, additive × additive epistasis interactions between the QTL were performed using the QTLNetwork (version 2.0) based on the mixed-model based composite interval mapping (MCIM) method. Positive additive effects indicated that alleles originating from Hongjiugu increased the phenotypic value, whereas negative additive effects indicated that alleles derived from Yugu18 increased the phenotypic value. QTL across different environments for the same trait were considered to be the same and were assigned the same name when the confidence intervals overlapped and the additive effects originated from the same parental line. The partially overlapping confidence intervals were merged and considered the final confidence intervals. QTL that explained more than 10% of phenotypic variation in at least one environment were considered major QTL. The QTL nomenclature was designated beginning with the letter “q”, followed by the trait abbreviation, the chromosome number and the QTL serial number.

### Candidate gene prediction

In this study, the QTL were considered a major and stable QTL consistently detected in at least two environments with R^2^ > 10%. The genomic regions of the major and stable QTL were used to predict the genes involved in hull colour formation. A list of genes and their gene annotations within the physical interval of these QTL were downloaded from the Phytozome database (https://phytozome-next.jgi.doe.gov/). Gene Ontology (GO) enrichment and the Kyoto Encyclopedia of Genes and Genomes (KEGG) analyses [41] were performed using OmicShare Tools (https://www.omicshare.com/tools/Home/Soft/pathwaygseasenior). Candidate genes related to hull colour were identified by combining the GO and KEGG annotation information of the genes and previous studies.

### Supplementary Information

**Additional file 1: Supplementary Table 1.** Data statistics of sequencing clean data of the parents and the RILs of the foxtail millet.

**Additional file 2: Supplementary Table 2.** Data statistics of genome coverage and mean depth.

**Additional file 3: Supplementary Table 3.** Detailed information of candidate genes in QTL genome regions annotated by KEGG and GO database.

## Data Availability

The raw sequence data generated during the current study are available in the publicly accessible National Center for Biotechnology Information (NCBI, https://www. ncbi.nlm.nih.gov/) database with accession number PRJNA915584.
